# Mg Alloy Matching Layers for Broadband PZT-5H/Epoxy 1-3 Composite Underwater Acoustic Transducers: Acoustic Characterization and Design Simulation

**DOI:** 10.3390/s26216972

**Published:** 2026-11-04

**Authors:** Ziao Xu, Fulin Wang, Qi Zhou, Zhijian Cai, Feifei Guo, Zengzhe Xi, Jie Dong

**Affiliations:** 1 National Engineering Research Center of Light Alloy Net Forming, State Key Laboratory of Metal Matrix Composite, School of Materials Science and Engineering, Shanghai Jiao Tong University, Shanghai 200240, China; 2 Center of Acoustic Functional Materials and Applications, School of Advanced Manufacturing Engineering, Nanjing University, Suzhou 215163, China; 3 Xi’an Structure-Function Materials International Science and Technology Cooperation Base, School of Materials and Chemical Engineering, Xi’an Technological University, Xi’an 710021, China

**Keywords:** underwater acoustic transducer, matching layer, magnesium alloy

## Abstract

The acoustic impedance mismatch between piezoelectric materials and water loads remains a bottleneck that severely hampers the advancement of underwater acoustic transducers. In this work, we propose a novel design that utilizes magnesium (Mg) alloy as the matching layer for PZT-5H/Epoxy 1-3 composite underwater acoustic transducers. To assess the suitability of Mg alloy as a matching-layer material and provide basic data for transducer design, the microstructural dependence of the elastic and acoustic properties is systematically established for solution-treated, extruded, and forged GWZ932 (Mg-9Gd-3Y-2Zn-0.5Zr, wt.%) alloys. It is determined that the elastic coefficients and acoustic performance of the three Mg alloys are essentially independent of grain size, second phases, and texture, whereas the acoustic attenuation coefficient exhibited an increasing trend with grain size at 20 MHz. The three alloys exhibit nearly identical acoustic impedances of 11.01 ± 0.03 MRayl (solution treated), 10.94 ± 0.02 MRayl (extruded), and 11.02 ± 0.03 MRayl (forged). The performance of PZT-5H/Epoxy 1-3 composite transducers with four different matching-layer designs, including single-matching-layer designs (Mg alloy or Al alloy) and dual-matching-layer designs (Mg alloy + polymer or Al alloy + polymer), is simulated using a finite element method (FEM). The results demonstrate that the transducer with the Mg alloy + polymer dual-matching-layer design exhibits both optimal transmitting voltage response (TVR) and receiving voltage sensitivity (RVS) bandwidths, with −6 dB transmit and receive bandwidths of 188.7 kHz and 156.6 kHz, respectively. These simulation results indicate that the Mg alloy + polymer dual-matching-layer design is promising for use in broadband piezoelectric underwater acoustic transducers. This work presents a novel Mg-alloy-based matching-layer scheme, offering a new choice for the matching-layer design of underwater acoustic transducers.

## 1. Introduction

Due to the complexity of the marine environment, underwater detection, tracking and recognition, primarily applied in submersibles, submarines, and scientific or surveillance missions, rely predominantly on acoustic waves [1]. Underwater acoustic transducers, which are capable of transmitting and receiving acoustic signals, serve as the core components of underwater detection, tracking and recognition systems [2,3]. Key evaluation parameters for these transducers include operating frequency, bandwidth, power density, and weight [4,5,6]. Compared with low-frequency acoustic signals, high-frequency signals offer superior resolution and stronger anti-interference capabilities [7,8]. To expand the information capacity of high-frequency acoustic signals, it is essential to enhance their bandwidth [9,10]. Consequently, the development of high-frequency, broadband underwater acoustic transducers is of urgent necessity for improving detection, tracking and recognition performance.

Piezoelectric underwater acoustic transducers currently dominate contemporary underwater detection, tracking and recognition systems, benefiting from their high electromechanical conversion efficiency, rapid response, and broad applicability [1,11]. High-frequency piezoelectric transducers operating above 100 kHz are typically constructed with an air-backed structure, incorporating piezoelectric materials and matching layers [9]. The development of underwater acoustic transducers has long been driven by advances in piezoelectric materials [3,12,13,14,15,16]. 1-3 piezoelectric composites possess lower acoustic impedance, higher thickness electromechanical coupling coefficients, and reduced mechanical quality factors, all of which contribute to the enhanced bandwidth and sensitivity of underwater acoustic transducers [17,18]. Moreover, the 1-3 composites can effectively suppress lateral modes, thereby yielding a purer thickness-mode vibration response [8]. Consequently, 1-3 piezoelectric composites are selected as the active elements in high-frequency underwater acoustic transducers. The 1-3 piezoelectric composite transducers commonly adopt a double-layer matching configuration. The first matching layer is typically fabricated from powdered metal-epoxy or metal oxide-epoxy composites, which cannot realize satisfactory impedance matching between 1-3 composites and a water medium [9,10]. Furthermore, they suffer from high acoustic attenuation coefficients, which severely degrade acoustic transmission efficiency and generate heat [19,20]. Bian et al. proposed an acoustic gradient impedance matching-layer material to enhance the bandwidth of the 1-3 piezoelectric composite transducers [8]. However, the fabrication process of the acoustic gradient impedance matching layer is overly complicated, which hinders its large-scale application. To date, developing matching-layer materials with suitable impedance for 1-3 composites remains a critical challenge.

In contrast to composite matching layers, light metals such as aluminum and titanium alloys are suitable for mass production and exhibit low acoustic attenuation coefficients, with acoustic impedances of approximately 17 and 22 MRayl, respectively. These metals have been widely employed as front masses (matching layers) for piezoelectric ceramic underwater acoustic transducers to enhance bandwidth and radiated power [21]. However, for 1-3 piezoelectric composite transducers, the acoustic impedance of titanium alloys is excessively high, precluding their use as matching layers, whereas the acoustic impedance of aluminum alloys is relatively close to that of the 1-3 composites but far from that of water, resulting in inadequate impedance matching. By comparison, aluminum alloys, magnesium alloys possess lower density and sound velocity, with an acoustic impedance of approximately 10 MRayl that enables favorable matching between the 1-3 piezoelectric composite and water, thereby improving acoustic transmission efficiency and broadening the bandwidth [22]. In our previous work, magnesium alloys were successfully implemented as the first matching layer in PMN-PT single crystal and PZT ceramic medical ultrasound transducers, leading to significant enhancements in both bandwidth and sensitivity [23,24,25]. Evidently, magnesium alloys as matching layers exhibit distinct advantages in enhancing the bandwidth and sensitivity of piezoelectric transducers. Furthermore, magnesium alloys exhibit superior mechanical properties, including high specific strength, high specific stiffness, and excellent damping capacity and thermal conductivity [26,27]. These attributes not only enable lightweight construction but also facilitate effective heat dissipation under high-power operation. The combination of high damping and good thermal conductivity helps suppress temperature rise induced by vibration energy dissipation, thereby enhancing the reliability and stability of the transducers. Nevertheless, the application of magnesium alloys as matching layers in underwater acoustic transducers remains largely unexplored. Underwater acoustic transducers differ from medical ultrasound transducers in piezoelectric materials, operating frequencies, and structural designs. This raises the critical question of whether magnesium alloys can achieve optimal matching performance in underwater acoustic applications—a question that requires systematic investigation.

Based on the demand for high-frequency, broadband, and high-power underwater acoustic transducers in sonar, detection, ranging, imaging, navigation, and acoustic sensing applications, this work employs 1-3 piezoelectric composites as the active element and GWZ932 magnesium alloy as the matching layer. First, the microstructure, mechanical properties, elastic properties, and acoustic attenuation characteristics of GWZ932 magnesium alloy were systematically investigated, providing fundamental data for the design of underwater acoustic transducers. Furthermore, single-matching-layer (Mg alloy) and double matching-layer (Mg alloy + Polymer) configurations for underwater acoustic transducers were designed via the finite element method (FEM), and their performance was systematically compared with that of Al alloy matching-layer transducers with identical structural design.

## 2. Elastic and Acoustic Properties of GWZ932 Magnesium Alloy

### 2.1. Microstructure of GWZ932 Magnesium Alloy

Compared with conventional commercial magnesium alloys, such as the AZ (Mg-Al-Zn) and Mg-RE (RE = Gd, Ho, Er) alloys, the GWZ932 (Mg-9Gd-3Y-2Zn-0.5Zr, wt.%) alloy forms long-period stacking ordered (LPSO) phases through the synergistic alloying of Gd, Y, and Zn. The high strength and stiffness of these LPSO phases, combined with their unique kinking deformation mechanism, contribute to a synergistic enhancement of both the strength and ductility of the GWZ932 alloys [28,29,30]. The processing methods of magnesium alloys substantially alter their microstructure, which in turn governs their elastic and acoustic attenuation properties. Therefore, this work investigates the microstructure of GWZ932 magnesium alloy processed by solution treatment, extrusion, and forging. The magnesium alloys used in this study are provided by the National Engineering Research Center of Light Alloy Net Forming, Shanghai Jiao Tong University. The actual elemental composition of the alloy determined by the Luoyang Institute of Special Materials Research is shown in Table 1. The solution treatment was conducted at 520 °C for 12 h, the extrusion was performed at 420 °C with an extrusion ratio of 1:9, and the forging was carried out at 450 °C with a single-pass reduction of 40%, five deformation passes, and a forging speed of 10 mm/s. Microstructural characterization was performed using scanning electron microscopy (SEM, TESCAN Mira3). The accelerating voltage was 20 kV. The data cleaning routine was performed using AZtecCrystal software (version 2.1).

**Figure 1:**
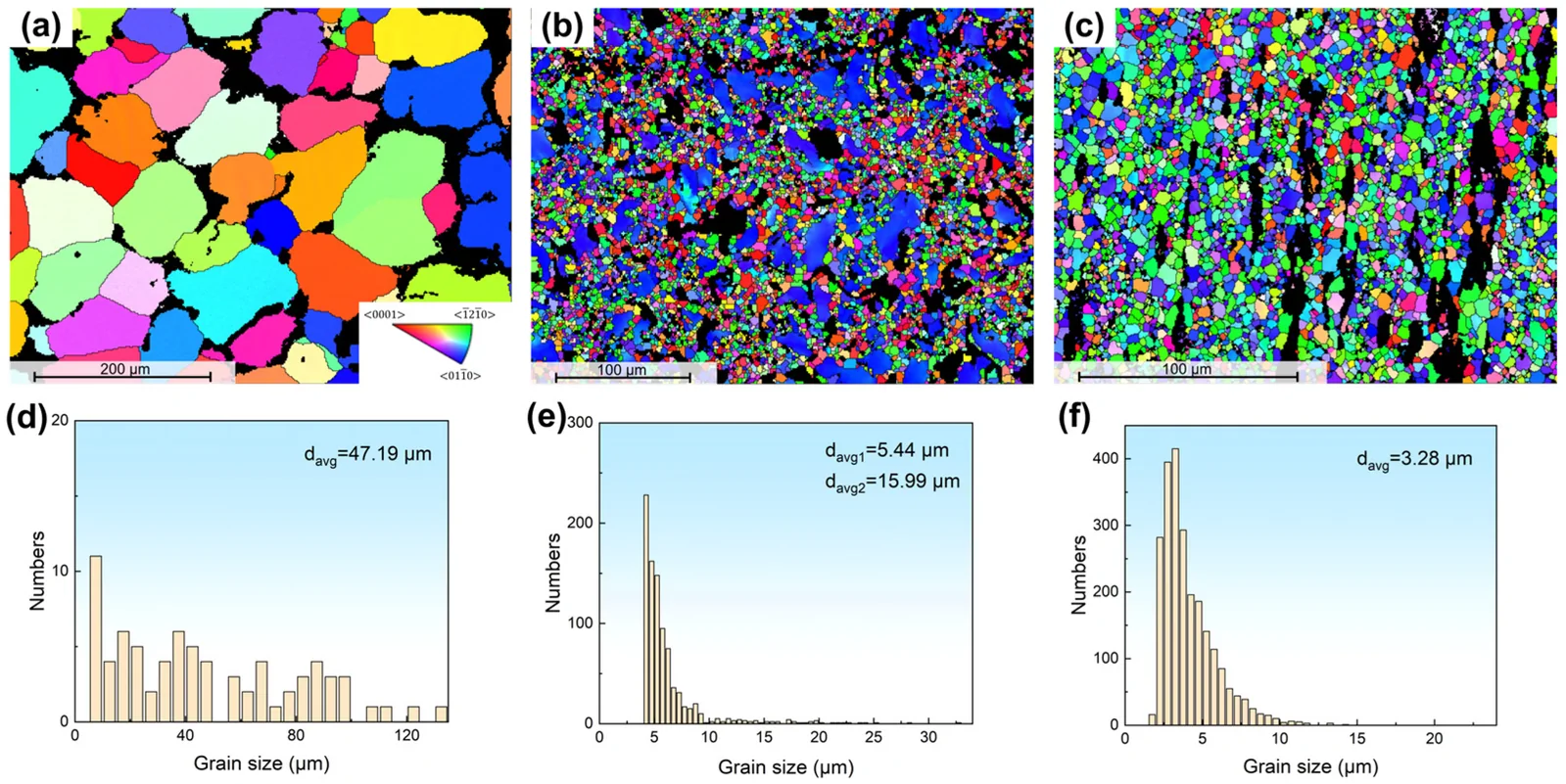
EBSD orientation and grain size distribution maps of the GWZ932 magnesium alloy in the solution-treated, as-extruded, and as-forged conditions. (**a**,**d**): solution-treated state; (**b**,**e**): as-extruded state; (**c**,**f**): as-forged state.

Electron backscatter diffraction (EBSD) was used to characterize the grain size and the distribution of the second phase in the GWZ932 magnesium alloy in three processing states. As shown in Figure 1a–c, the solution-treated GWZ932 alloy is characterized by a coarse-grained microstructure, with grain sizes ranging from a few tens of micrometers to more than 100 μm. The scanning step size was 1.5 μm, the mapped area contained 381 × 286 pixels, the number of grains was 76, and the indexing rate was 79.93%. The extruded GWZ932 alloy exhibits a bimodal microstructure, consisting of fine dynamically recrystallized grains and coarse grains. The scanning step size was 1.2 μm, the mapped area contained 314 × 235 pixels, the number of grains was 1466, and the indexing rate was 71.04%. The forged GWZ932 alloy is primarily composed of fine grains with sizes below 10 μm. The scanning step size was 0.5 μm, the mapped area contained 458 × 339 pixels, the number of grains was 2673, and the indexing rate was 72.57%. The grain size distributions characterized by the equivalent circle diameter are presented in Figure 1d–f. The solution-treated GWZ932 alloy exhibits an average grain size of 47.19 μm, with a maximum of 130.96 μm. For the extruded GWZ932 alloy, the proportion of grains larger than 10 μm markedly decreases. Setting 10 μm as the threshold between fine and coarse grains, the average grain sizes for the two fractions are determined to be 5.44 μm and 15.99 μm, respectively. The forged GWZ932 alloy shows an average grain size of 3.28 μm.

**Figure 2:**
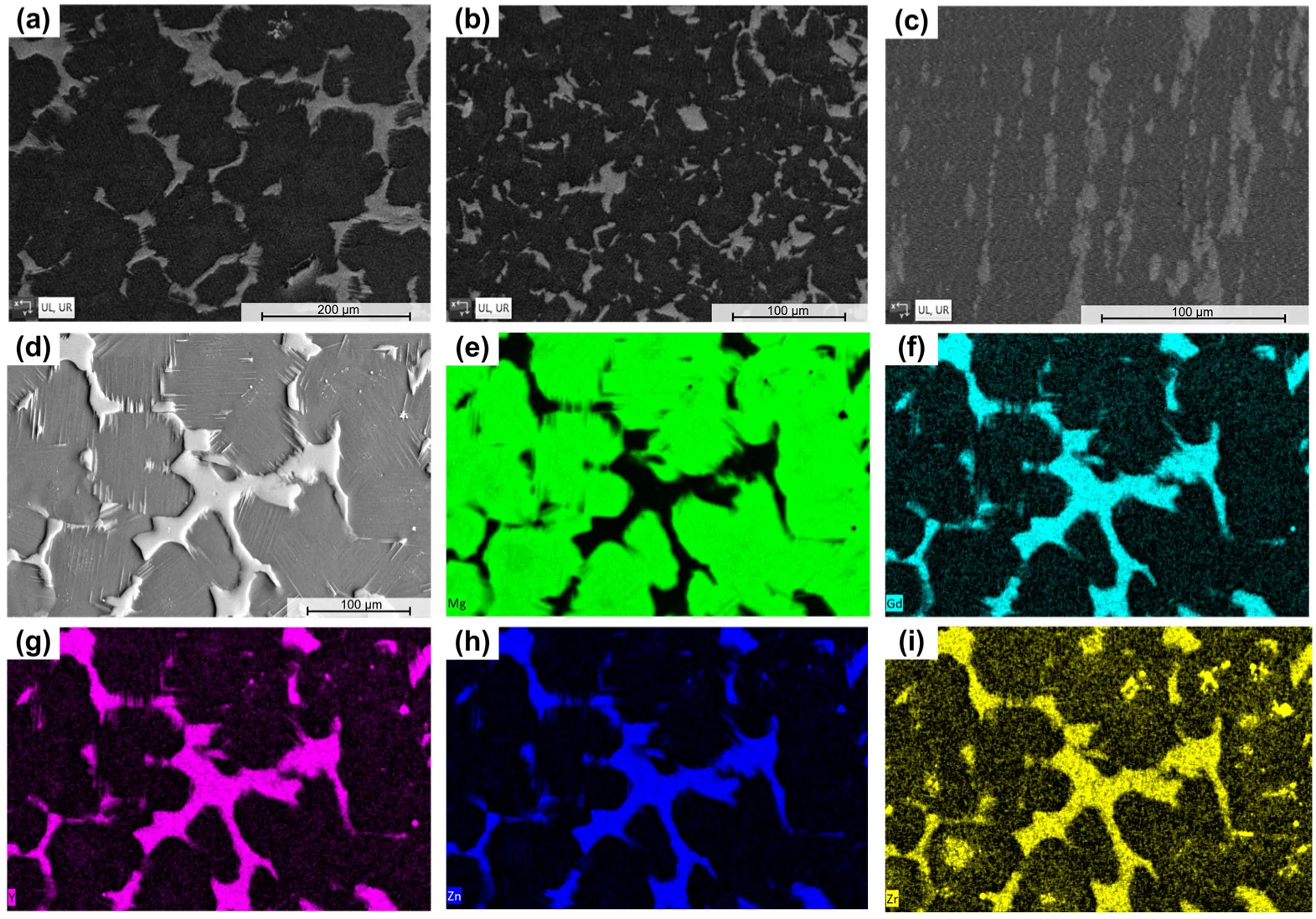
FSD maps of the GWZ932 magnesium alloy (**a**) in the solution-treated, (**b**) as-extruded, and (**c**) as-forged conditions. (**d**) BSE image and (**e**–**i**) EDS element maps of Mg, Gd, Y, Zn and Zr.

Furthermore, it should be noted that the block-shaped unresolved regions in Figure 1a–c correspond to the second phase (LPSO phase). To reveal the LPSO phase distribution more clearly, forward scattered electron detector (FSD) images were acquired. As shown in Figure 2a–c, the dark regions represent the GWZ932 matrix and the bright regions represent the LPSO phase. For verification, backscattered electron (BSE) images were acquired and energy-dispersive X-ray spectroscopy (EDS) mapping was performed on selected regions of the corresponding material in Figure 2a. The results are shown in Figure 2d–i. It can be observed that the dark regions in the BSE image are dominated by Mg, while the bright regions mainly consist of Gd, Y and Zn. This indicates that the bright regions correspond to the LPSO phase formed by the synergistic alloying of Gd, Y and Zn. In the solution-treated GWZ932 alloy, the second phase shows an irregular blocky morphology, predominantly distributed at grain boundaries. In the extruded GWZ932 alloy, the second phase is fragmented and dispersed, indicating that the original LPSO phase underwent deformation during extrusion, transforming from a blocky to a fragmented morphology. In the forged GWZ932 alloy, the second phase exhibits an elongated morphology with a distinct directional alignment, indicating that the LPSO phase was continuously compressed and deformed along the forging direction. The second-phase fractions quantitatively analyzed by AZtecCrystal software are 16.3%, 24.5%, and 23.0% for the solution-treated, extruded, and forged states, respectively.

**Figure 3:**
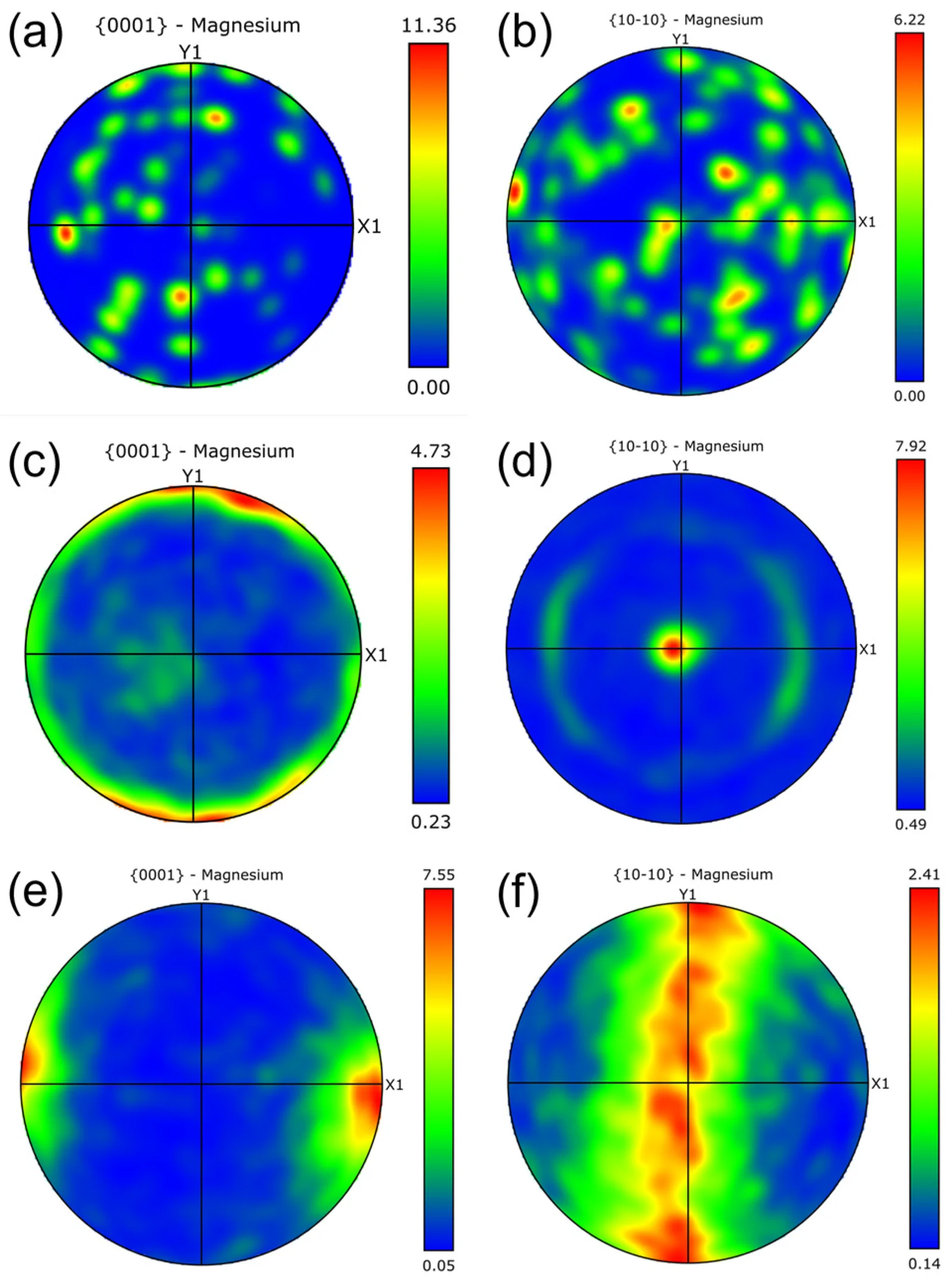
Pole figures of the GWZ932 magnesium alloy in the solution-treated, as-extruded, and as-forged conditions along the {0001} and {10-10} directions. (**a**,**b**) Solution-treated state; (**c**,**d**): as-extruded state and; (**e**,**f**): as-forged state.

The crystal orientation distributions of the three GWZ932 magnesium alloys were obtained via EBSD characterization. The texture of the solution-treated GWZ932 alloy is shown in Figure 3a,b. Although it shows the maximum texture intensity, the high-intensity poles are uniformly distributed on both {0001} and {10-10} planes, so it can still be considered a random texture. The texture of the extruded GWZ932 alloy is presented in Figure 3c,d. An obvious ring-like distribution is formed at the periphery of the pole figure on {0001} plane and the {10-10} pole figure exhibits a central intensity maximum, which corresponds to the typical basal texture of extruded Mg alloys. The texture of the forged GWZ932 alloy is shown in Figure 3e,f. It can be seen that the {0001} intensity maxima split into two separated clusters on the equator. Meanwhile, the maximum pole density of the {10-10} plane is only 2.41. Although the maximum pole density of the forged sample on {0001} reaches 7.55, these high-intensity poles are spatially dispersed rather than concentrated in a single direction. Consequently, the forged alloy exhibits a near-random texture with much weaker anisotropy. Overall, the solution-treated and forged alloys exhibit relatively weak texture, which does not exert a significant effect on the anisotropy of the material. In contrast, the extruded alloy has a higher texture intensity, and its impact on anisotropy requires further investigation.

### 2.2. Elastic Property of GWZ932 Magnesium Alloy

The crystal structure of the GWZ932 magnesium alloy at room temperature is hexagonal close-packed (HCP), with a macroscopic symmetry belonging to the 6/mmm point group [31,32,33]. The elastic properties of GWZ932 alloys are described by the elastic stiffness coefficients *c*_ij_ and elastic compliance coefficient *s*_ij_. The coefficients *c*_ij_ and *s*_ij_ can be achieved using the self-consistent full-matrix technique [23]. The dimensions of GWZ932 specimens used for elastic coefficient measurements are 15 mm//[100]_C_ × 15 mm//[010]_C_ × 5 mm//[001]_C_. [100]_C_, [010]_C_ and [001]_C_ axes are designated as directions 1, 2, and 3, respectively. For the extruded and forged GWZ932 specimens, direction 3 is parallel to the extrusion direction for the extruded specimen and to the compression direction for the forged specimen.

**Figure 4:**
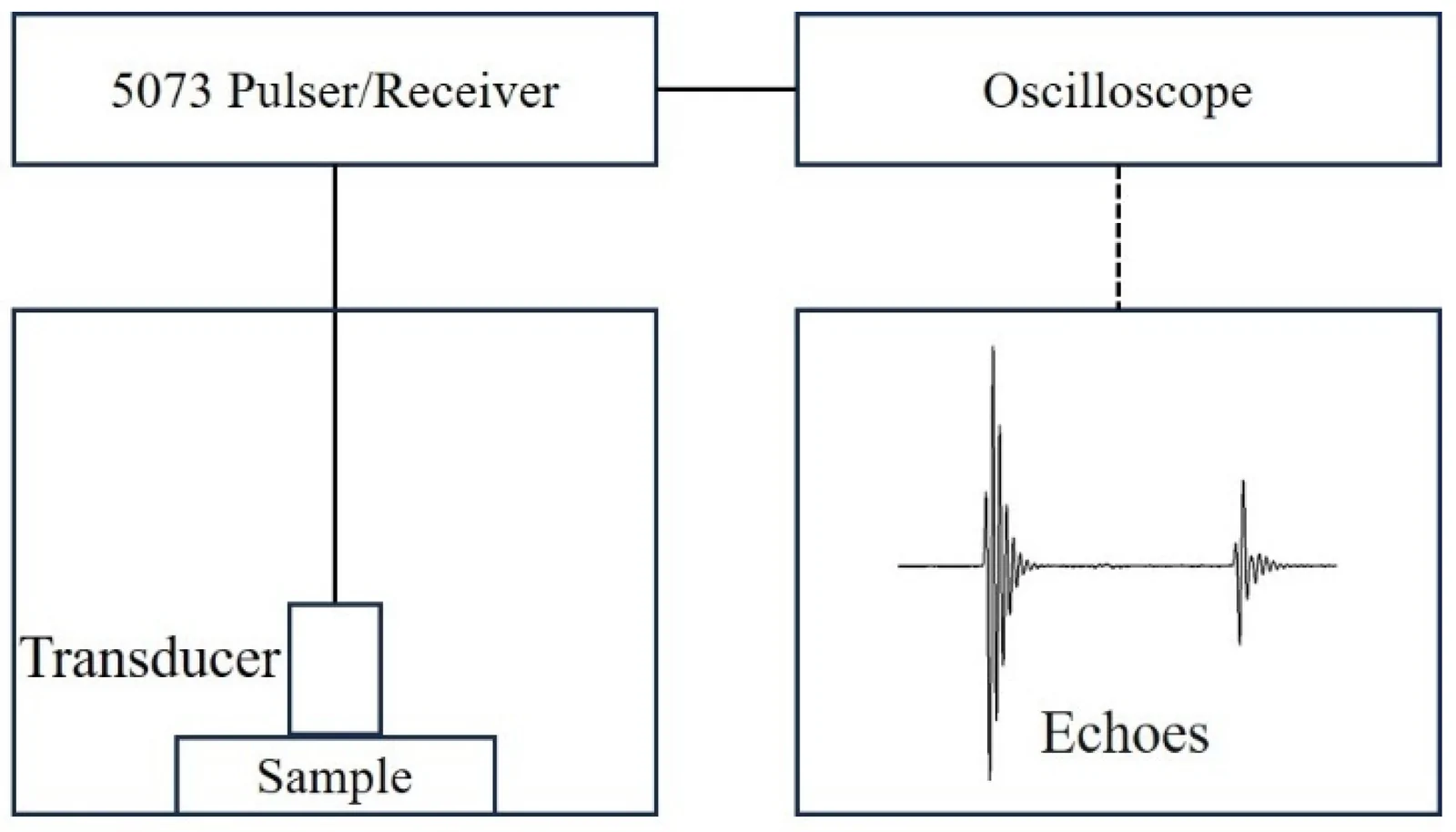
Schematic diagram of the ultrasonic pulse-echo measurement setup.

The elastic stiffness coefficient *c* of GWZ932 specimens can be calculated through *c = ρv*^2^, where *ρ* is the density and *v* is the sound velocity. The density *ρ* was measured using the Archimedes method, and the sound velocity *v* was measured using the ultrasonic pulse-echo method. A schematic diagram of the ultrasonic pulse-echo setup is shown in Figure 4. Pulse signals from a generator drive the ultrasonic transducer to emit waves that propagate through the specimen. The echoes reflected from the specimen surfaces are received by the transducer, converted to electrical signals, and displayed on an oscilloscope. Three separate specimens were tested for each of the three processing conditions of the GWZ932 alloys (nine specimens in total). Density and ultrasonic pulse-echo measurements were each repeated three times on every specimen. The sound velocity of the magnesium alloy was calculated from the time difference between successive echoes and the thickness of the specimen.

The elastic stiffness coefficients *c*_11_, *c*_33_, *c*_44_ and *c*_66_ of the GWZ932 alloy were obtained by measuring the sound velocities in different directions. Specifically, *c*_11_ and *c*_66_ were derived from the longitudinal wave velocity *v*_l_^[100]^ and shear wave velocity *v*_s_^[100]^ measured in the [100]_C_ direction, while *c*_33_ and *c*_44_ were derived from the longitudinal wave velocity *v*_l_^[001]^ and shear wave velocity *v*_s_^[001]^ measured in the [001]_C_ direction. Other elastic coefficients were calculated using the formulas from our previous work [23].

The measured densities and sound velocities of the three GWZ932 alloys are listed in Table 2. It can be observed that the forged GWZ932 alloy exhibits a slightly higher density than its solution-treated and extruded counterparts, suggesting that the forging process reduces residual porosity within the material to some extent. The longitudinal and shear wave velocities of three alloys are approximately 5580 m/s and 3000 m/s, respectively. The elastic stiffness coefficients *c*_11,_ *c*_33,_ *c*_44,_ and *c*_66_ of the three GWZ932 alloys were calculated using the measured densities and longitudinal- and shear-wave velocities, as shown in Table 3. The remaining stiffness coefficients and the elastic compliance coefficients subsequently derived from these four coefficients are summarized in Table 4. Taking the extruded GWZ932 alloy as an example, it can be seen that the differences between *c*_11_ and *c*_33_, *c*_12_ and *c*_13_, and *c*_44_ and *c*_66_ are negligible. Similar trends are observed in the compliance coefficients *s_ij_*. The other two GWZ932 alloys also exhibited similar trends in the elastic stiffness and compliance coefficients. These results indicate that the elastic anisotropy of the three GWZ932 alloys is not significant.

### 2.3. Acoustic Impedance and Attenuation of GWZ932 Magnesium Alloy

**Figure 5:**
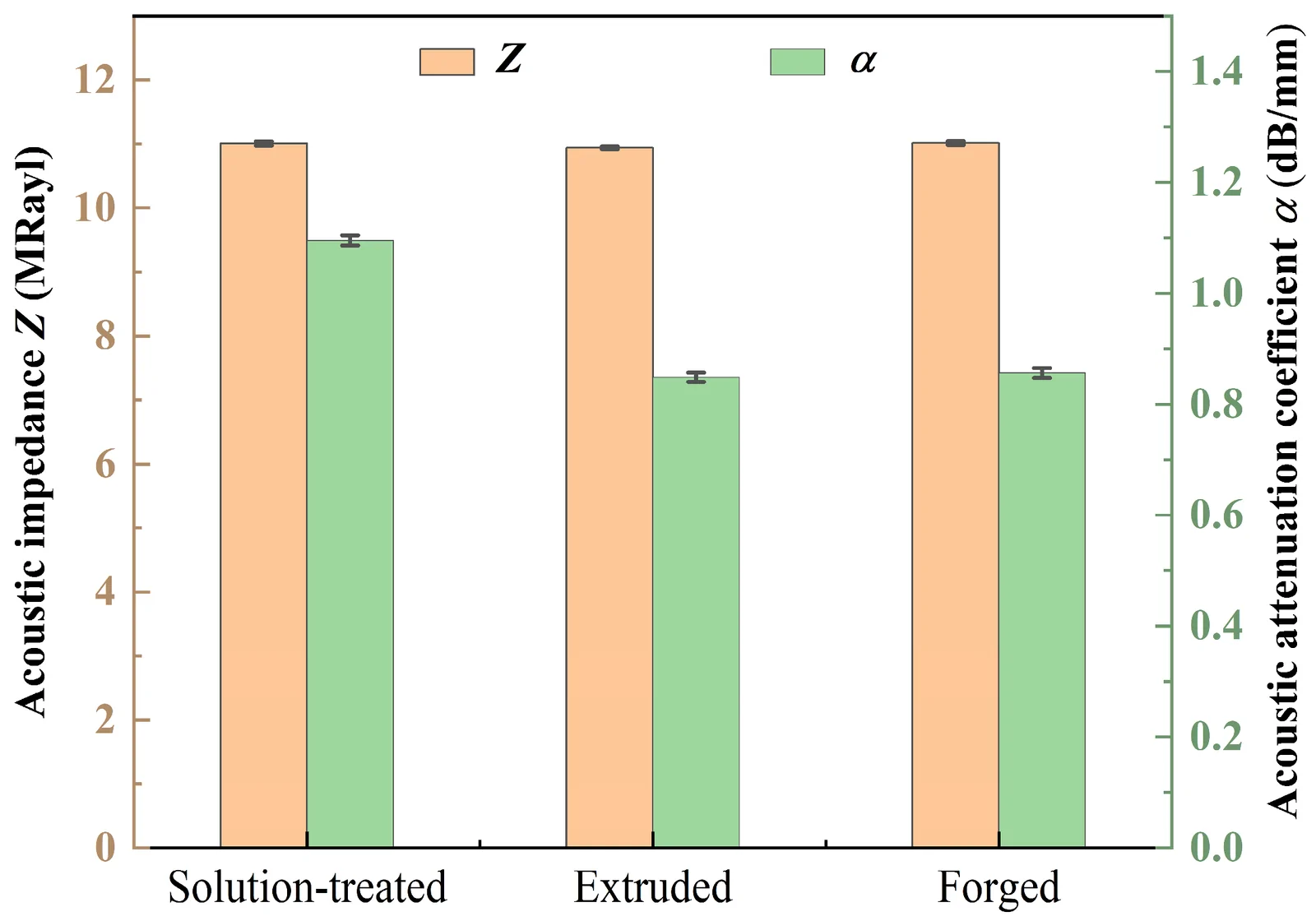
Acoustic impedance and acoustic attenuation coefficient @ 20 MHz of the GWZ932 magnesium alloy in the three different states (solution-treated, extruded, and forged).

The acoustic impedance of the GWZ932 alloys can be calculated from density and sound velocity using the equation *Z* = *ρv*. Since the sound velocity can be considered isotropic, the acoustic impedance can be regarded as independent of crystallographic direction. The acoustic impedance of the three GWZ932 alloys was calculated using *v*_l_[001]. As shown in Figure 5, the three GWZ932 alloys exhibit nearly identical acoustic impedances: 11.01 ± 0.03 MRayl (solution-treated), 10.94 ± 0.02 MRayl (extruded), and 11.02 ± 0.03 MRayl (forged). These values are lower than the ~17 MRayl of aluminum alloys.

As the matching-layer of underwater acoustic transducers, the acoustic attenuation coefficient is also an important factor affecting sound transmission efficiency. The longitudinal-wave acoustic attenuation coefficients *α* of the three GWZ932 alloys measured at 20 MHz are presented in Figure 5. The *α* values of the solution-treated, extruded, and forged GWZ932 alloys are 1.095 ± 0.009 dB/mm, 0.849 ± 0.008 dB/mm, and 0.857 ± 0.009 dB/mm at 20 MHz, respectively. At this frequency, the wavelength is of the same order as the dimensions of scattering centers such as grain boundaries and second-phase particles, so the attenuation is predominantly attributed to stochastic scattering [34]. Thus, the higher attenuation coefficient of the solution-treated alloy is primarily attributed to its large grain size.

The above results suggest that the three GWZ932 alloys exhibit essentially isotropic elastic behavior, which is independent of material texture. Consequently, the cutting direction can be neglected when machining the matching layers. Furthermore, the acoustic impedances of the three alloys are essentially identical. Considering that the GWZ932 magnesium alloy exhibits a more favorable acoustic impedance than that of aluminum alloy, a widely used matching-layer material, it can be used as a matching-layer material for transducer fabrication. In this study, the extruded GWZ932 alloy was selected as the matching-layer material for the 1-3 composite underwater transducer design.

## 3. Transducer Design and Analysis Based on FEM Model

**Figure 6:**
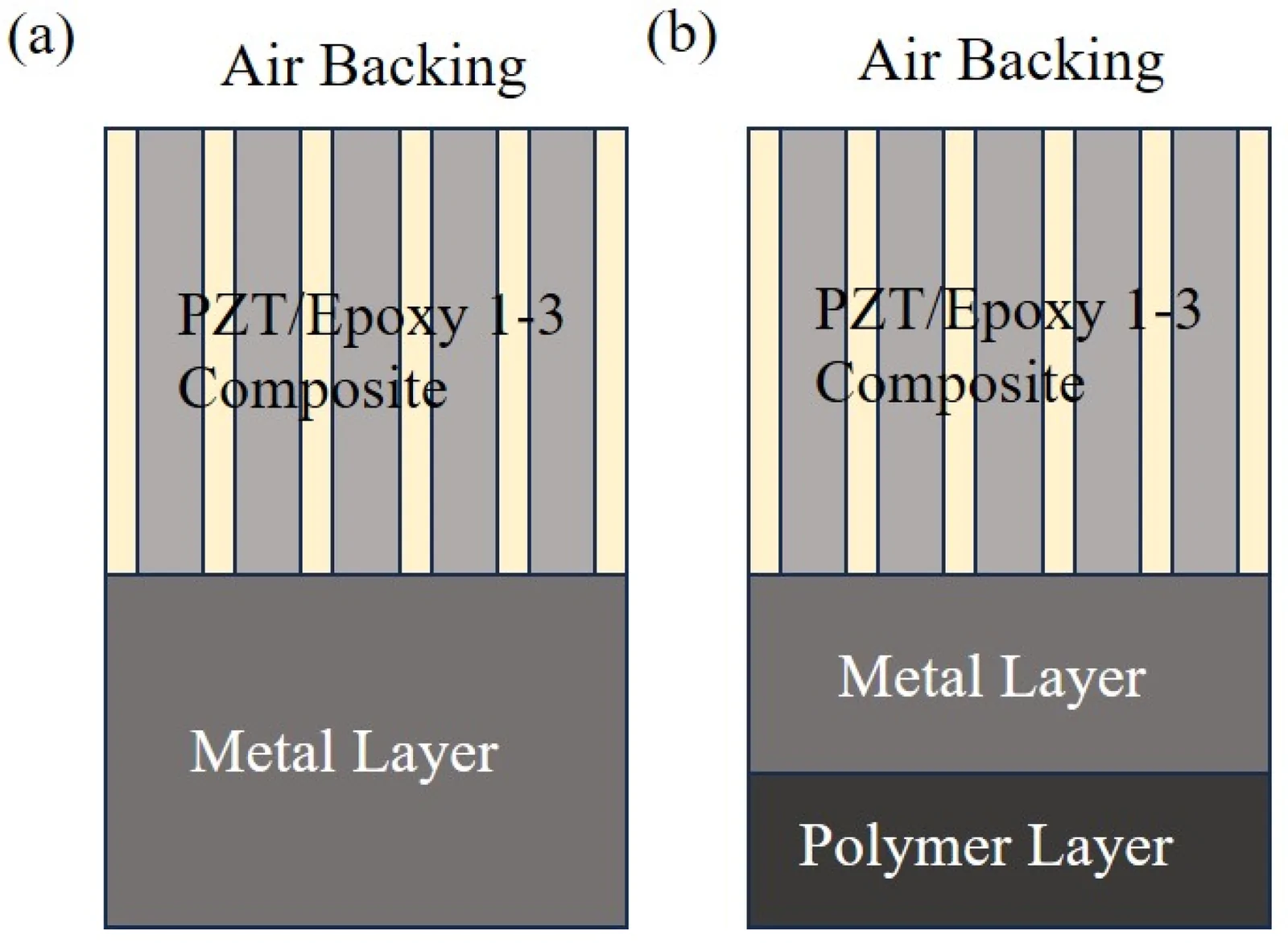
Structural diagrams of the four transducers: (**a**) single -matching layer (Mg alloy or Al alloy) and (**b**) dual matching layers (Mg alloy + polymer or Al alloy + polymer).

**Figure 7:**
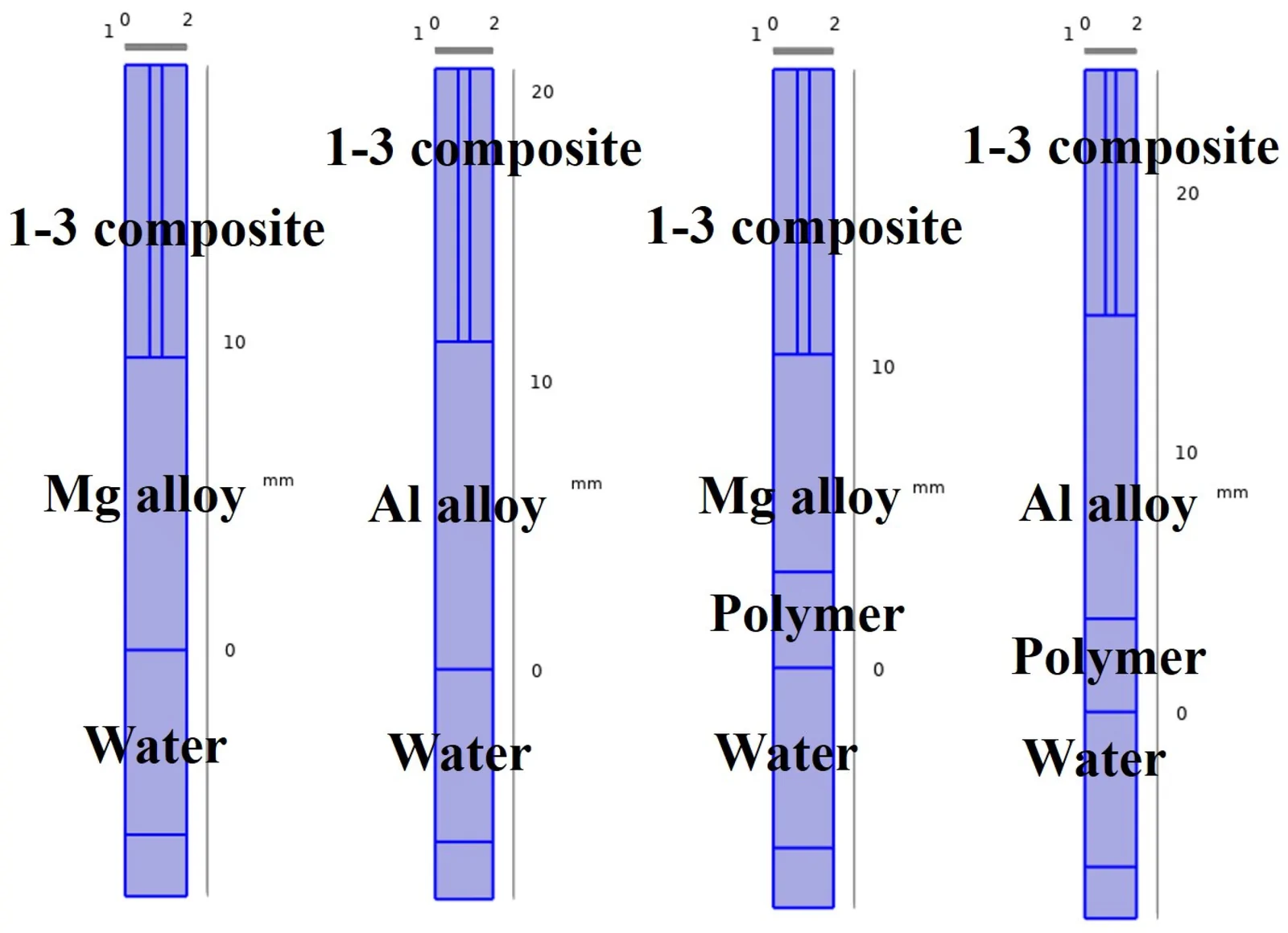
Periodic unit cell model of the four 1-3 piezoelectric composite transducer for finite element method simulation diagram.

Four transducers with different matching-layer configurations were designed. Figure 6 presents the structural configurations of the four transducers, encompassing single-matching-layer designs (Mg alloy or Al alloy) and dual-matching-layer designs (Mg alloy + polymer or Al alloy + polymer). The design of the 1-3 composite underwater acoustic transducer was carried out using a three-dimensional repeating unit cell model using the finite element method (FEM) simulation. The model uses the Solid Mechanics, Electrostatics, and Pressure Acoustics (Frequency Domain) interfaces in COMSOL Multiphysics 6.4, and realizes the interaction among the structural, electric, and acoustic fields through piezoelectric coupling and acoustic-structure coupling. The symmetric boundary conditions are imposed on the four lateral surfaces of the unit cell to approximate the operating condition of an infinite planar transducer. The water load is implemented through an explicit water domain and acoustic-structure coupling. The normal water domain has a thickness of 6 mm along the propagation direction with a 2 mm-thick Perfectly Matched Layer (PML) on the outer side to simulate an open acoustic domain. The model adopted ideal conditions, without introducing material losses or empirical damping. Free tetrahedral meshes were used for the solid and the normal water domain, with the maximum mesh size set to one-fifth of the wavelength at a reference frequency of 220 kHz. A swept mesh is used for the PML, with the maximum element size set to one-seventeenth of the reference wavelength. This model focuses on longitudinal vibration along the thickness direction to compare the effects of using Mg and Al alloys as the matching layer on the transmitting voltage response (TVR), receiving voltage sensitivity (RVS), and bandwidth. For the TVR calculation, a 1 V terminal voltage is used, and the far-field sound pressure level is reported at 1 m along the axis of the model. For the RVS calculation, a 1 Pa normally incident plane wave is employed, and the receiving voltage sensitivity is obtained from the open-circuit terminal voltage. All calculations were finally carried out over the range of 80–300 kHz with a step size of 0.1 kHz. As this study focuses on the advantages of magnesium alloy as a matching layer for 1-3 composite underwater transducers, a complete finite-dimension transducer model is not constructed. It should be noted that this idealized model neglects factors such as finite aperture, edge effects, packaging, and practical assembly. The results derived from this model are therefore intended to evaluate the material matching performance and cannot be directly equated with the performance of an actual transducer. Figure 7 shows the finite element models of the four transducers. The 1-3 composite consists of PZT-5H and epoxy resin. During the modeling process, the material parameters of PZT-5H, epoxy resin and the polymer matching layer shown in Table 5 were taken from the built-in COMSOL database, while the elastic parameters for Mg alloy and Al alloy are listed in Table 4.

**Figure 8:**
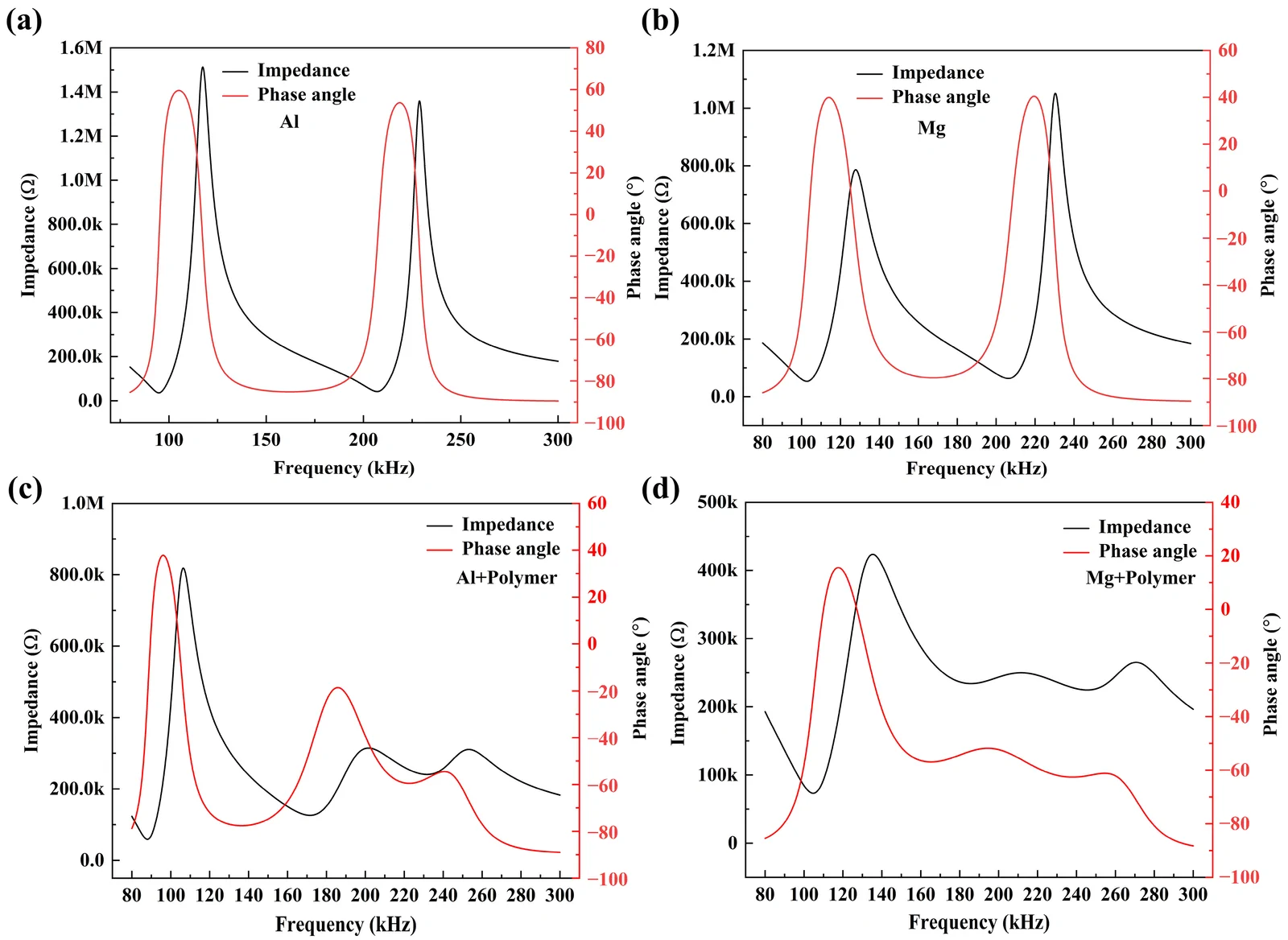
Simulated impedance and phase angle versus frequency curves for the four transducer designs: (**a**) Al alloy matching layer; (**b**) Mg alloy matching layer; (**c**) Al alloy+polymer matching layer; (**d**) Mg alloy+polymer matching layer.

The lateral dimensions of the minimum repeating unit are 2 mm × 2 mm and consists of a 1.6 mm × 1.6 mm PZT-5H pillar and a 0.4 mm kerf. The optimized axial dimensions (thicknesses) of the four transducer structures are provided in Table 6. The designed frequency of all four transducers is 200 kHz. The layer thickness optimization aims to enhance the broadband transmit/receive response under water loading. The initial dimensions were determined based on the half-wavelength thickness vibration and quarter-wavelength matching conditions, with the matching-layer thickness optimized via a parametric sweep. For the single-matching-layer structure, only the metal-layer thickness was swept, whereas for the double-matching-layer structure, the metal-layer and polymer-layer thicknesses were swept jointly. The final design was determined from a comparison of the continuous −6 dB bandwidths of the TVR and RVS, together with the overlap of their frequency bands, peak values, and in-band dips. For comparison, all four designs used identical 1-3 composite elements, poling and electrode configurations, side symmetry boundary conditions and water-loading condition, with only the matching-layer design parameters varied. The thickness of each layer was expressed in terms of the longitudinal wavelength at a frequency *f* of 200 kHz: *λ* = *c/f*, where λ and c are the longitudinal wavelength and sound velocity of the matching-layer materials, respectively. For the single-matching-layer design, the thicknesses of the Al alloy and Mg alloy layers are 0.371λ and 0.338λ, respectively. For the dual-matching-layer design, the thicknesses of the Al alloy and Mg alloy layers are 0.382λ and 0.257λ, and the corresponding polymer layers are 0.232λ and 0.241λ thick, respectively. Figure 8 presents simulated impedance and phase angle versus frequency curves for the four transducer designs. As observed, the transducers with a single matching layer of Al alloy or Mg alloy exhibit two well-separated and independent groups of resonance peaks, demonstrating the inadequacy of a single matching layer for achieving broad bandwidth transmission. For the dual-matching-layer transducers, three resonance peaks are generated in each configuration: the Al alloy + polymer configuration at 88 kHz, 171.5 kHz, and 231.7 kHz; and the Mg alloy + polymer configuration at 104.7 kHz, 185.8 kHz, and 245.5 kHz. It is evident that the Al alloy + polymer transducer exhibits a pronounced valley between the first and second resonance peaks. This may be attributed to the excessively high acoustic impedance of the Al alloy, which prevents effective coupling between the two resonances. By contrast, the impedance spectrum of the Mg alloy + polymer transducer is flat, indicating that the Mg alloy is effectively matched to the 1-3 composite in dual-matching-layer configuration, thereby enabling broadband transmission and reception.

**Figure 9:**
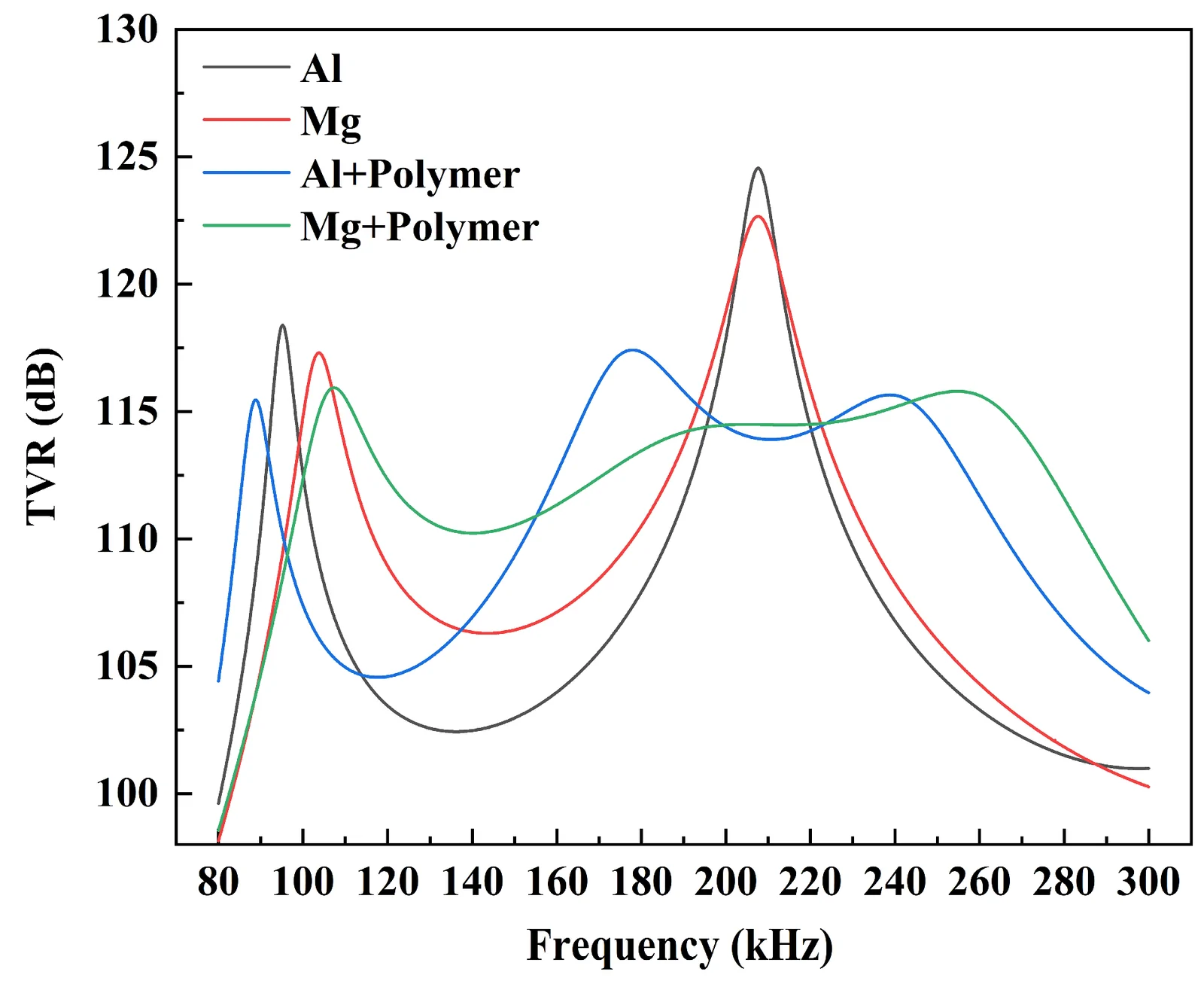
Simulated transmitting voltage response (TVR) spectra as a function of frequency for the four transducers.

Figure 9 presents the simulated transmitting voltage response (TVR) as a function of frequency for the four transducers. It can be observed that the transducers with a single matching layer of Al alloy or Mg alloy show a very narrow transmit bandwidth. For the dual-matching-layer transducers, both the Al alloy + polymer and the Mg alloy + polymer designs exhibit a significant improvement in transmit bandwidth. For the Al alloy + polymer transducer, the maximum TVR is 117.4 dB at 117.9 kHz, and the −6 dB transmit bandwidth is 104.2 kHz. By comparison, the Mg alloy + polymer transducer exhibits a flatter TVR curve, with a maximum TVR of 115.9 dB at 107.4 kHz. Its −6 dB bandwidth extends from 97.0 kHz to 285.7 kHz, corresponding to a transmit bandwidth of 188.7 kHz, which is wider than that of the counterpart. In addition, the thickness of the magnesium alloy matching layer is significantly smaller than that of the aluminum alloy matching layer, which effectively reduces the weight of the transducer and contributing to a lightweight design.

**Figure 10:**
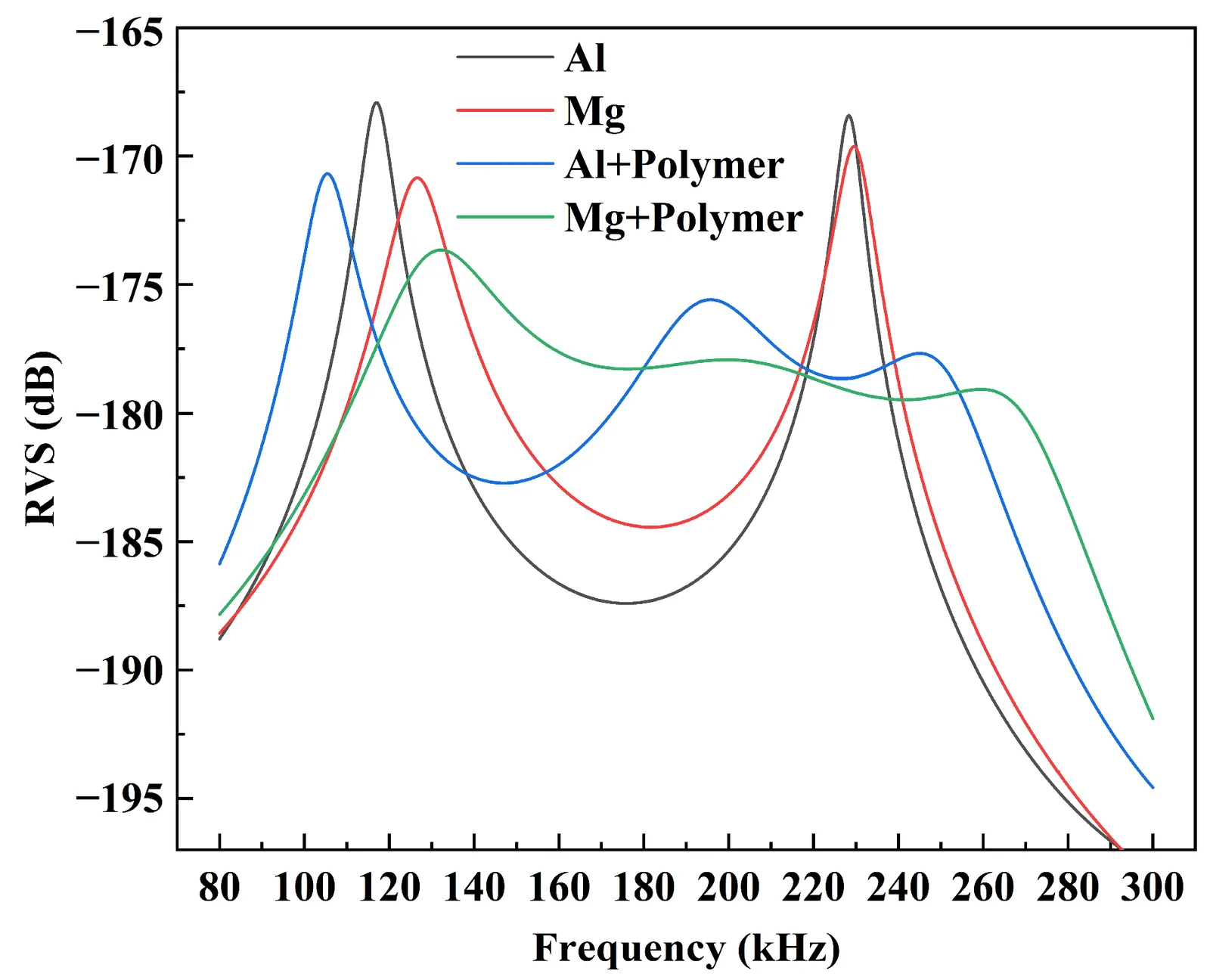
Simulated receiving voltage sensitivity (RVS) spectra as a function of frequency for the four transducers.

Figure 10 presents the simulated receiving voltage sensitivity (RVS) spectra as a function of frequency for the four transducers. Similar to the TVR results, the transducers with a single matching layer show a much narrower receive bandwidth than that of the dual-matching-layer transducers. For the Al alloy + polymer transducer, the maximum RVS value is −175.6 dB at 195.7 kHz, and the −6 dB receive bandwidth is 96.9 kHz. By comparison, the Mg alloy + polymer transducer exhibits a flatter RVS curve, with a maximum RVS of −173.7 dB at 132.1 kHz and a −6 dB receive bandwidth of 156.6 kHz, much wider than that of the Al alloy + polymer counterpart.

**Figure 11:**
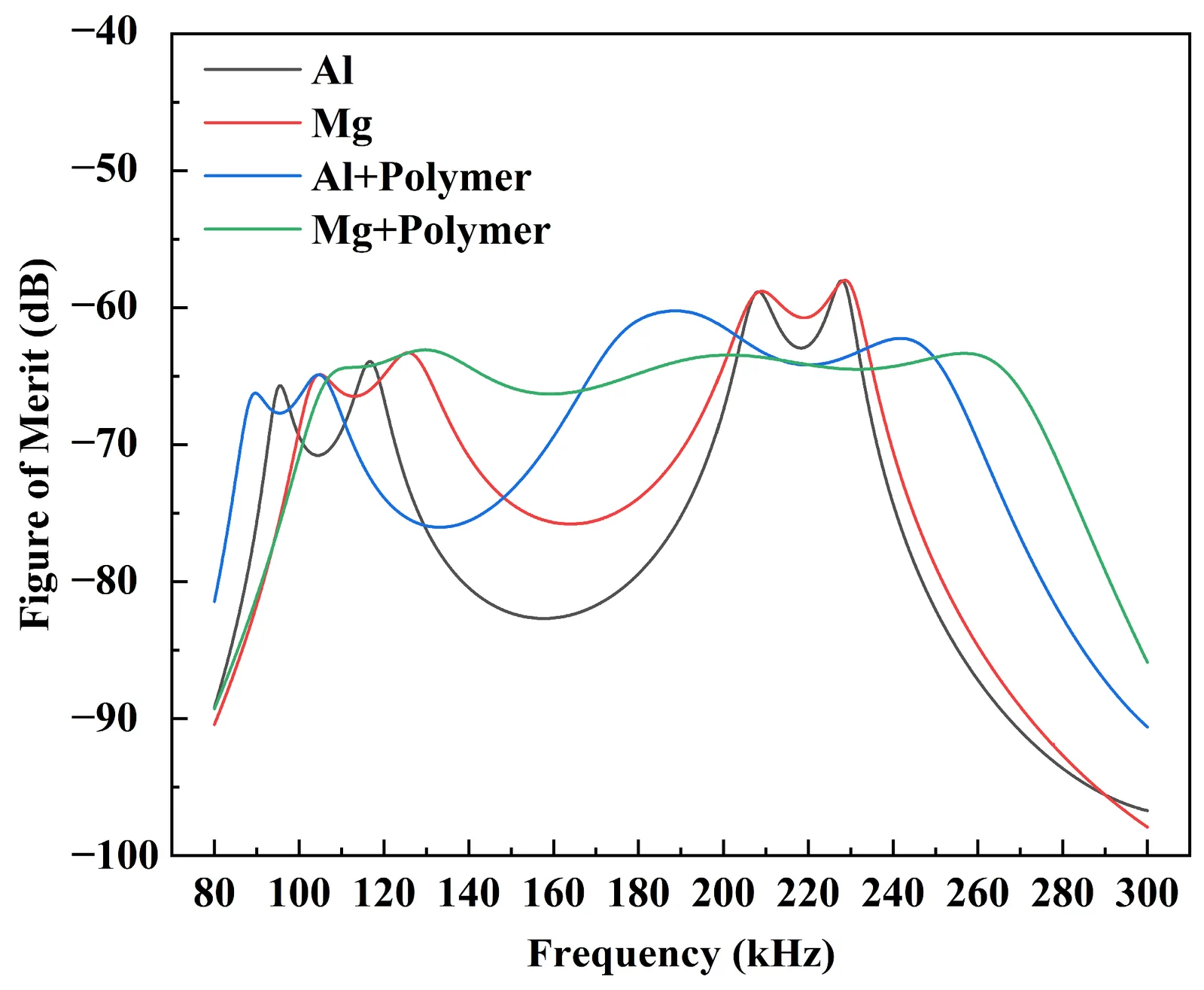
Simulated Figure of Merit (FOM) spectra as a function of frequency for the four transducers.

The combined sensitivity of the transmit and receive modes (TVR + RVS), i.e., the Figure of Merit (FOM), is used to evaluate the overall electroacoustic performance of underwater acoustic transducers, including their combined broadband performance, and uniformity within the operating frequency band. Figure 11 shows the FOM result of the four transducers. Obviously, compared to Al alloy, Mg alloy, and Al alloy + polymer transducers, the Mg alloy + polymer transducer exhibits a flatter FOM spectrum over a wide frequency range with a −6 dB bandwidth of 173.9 kHz. This result indicates the Mg alloy + polymer dual-matching-layer design can effectively flatten the combined sensitivity and broaden the −6 dB bandwidth.

## 4. Conclusions

In this work, the suitability of GWZ932 magnesium alloy as a matching-layer material for PZT-5H/Epoxy 1-3 composite underwater acoustic transducers was systematically assessed through material characterization and multiphysics numerical simulation. It is found that the elastic properties and acoustic impedance are insensitive to the microstructure and crystallographic orientation, whereas the acoustic attenuation coefficient increases with grain size. The acoustic impedances of the solution-treated, extruded, and forged GWZ932 alloys are 11.01 ± 0.03, 10.94 ± 0.02, and 11.02 ± 0.03 MRayl, respectively. Four PZT-5H/Epoxy 1-3 composite transducers with different matching layer designs—including single-matching-layer designs (Mg alloy or Al alloy) and dual-matching-layer designs (Mg alloy + polymer or Al alloy + polymer)—were modeled using the finite element method (FEM). The impedance spectra, transmitting voltage response (TVR), receiving voltage sensitivity (RVS) and Figure of Merit (FOM) demonstrate that the Mg alloy + polymer transducer exhibits superior performance compared to the other three transducers. Its −6 dB transmit and receive bandwidths reach 188.7 kHz and 156.6 kHz, respectively, indicating that Mg alloys are promising for improving the bandwidth of piezoelectric underwater acoustic transducers.

## Figures and Tables

**Table 1:** The elemental composition of the GWZ932 magnesium alloy.

Material	Elements and Contents (%)			
	Gd	Y	Zn	Zr
GWZ932 Mg alloy	8.93	3.11	1.98	0.45

**Table 2:** Density and sound velocity of the GWZ932 magnesium alloy under different treatment conditions.

Materials	*ρ* (kg/m^3^)	*v*_l_^[100]^ (m/s)	*v*_l_^[001]^ (m/s)	*v*_s_^[001]^ (m/s)	*v*_s_^[100]^ (m/s)
Solution-treated Mg alloy	1965.6 ± 1.2	5555.8 ± 16.9	5598.5 ± 17.0	2825.8 ± 45.4	3016.7 ± 59.1
Extruded Mg alloy	1964.5 ± 1.2	5570.7 ± 10.4	5571.9 ± 14.3	2761.4 ± 45.7	2893.4 ± 78.7
Forged Mg alloy	1977.1 ± 3.3	5537.3 ± 14.2	5572.4 ± 16.2	2825.8 ± 39.0	3000.0 ± 52.5

**Table 3:** Measured elastic constants of the three GWZ932 magnesium alloys (solution-treated, extruded and forged).

Materials	*c*_11_	*c*_33_	*c*_44_	*c*_66_
Solution-treated Mg alloy	6.08 ± 0.04	6.16 ± 0.04	1.57 ± 0.05	1.79 ± 0.07
Extruded Mg alloy	6.10 ± 0.03	6.10 ± 0.02	1.50 ± 0.05	1.65 ± 0.09
Forged Mg alloy	6.06 ± 0.04	6.14 ± 0.03	1.58 ± 0.04	1.78 ± 0.06

**Table 4:** Elastic constants of the three GWZ932 magnesium alloys (solution-treated, extruded and forged) and 2024 aluminum alloy.

	Elastic Stiffness Coefficient *c_ij_* (10^10^ N∙m^−2^)						Elastic Compliance Coefficient *s_ij_* (10^−12^ m^2^∙N^−1^)					
Materials	*c*_11_	*c*_12_	*c*_13_	*c*_33_	*c*_44_	*c*_66_	*s*_11_	*s*_12_	*s*_13_	*s*_33_	*s*_44_	*s*_66_
Solution-treated	6.08	2.5	2.23	6.16	1.57	1.79	21.12	−6.74	−5.20	20	63.69	55.87
Extruded	6.10	2.82	2.79	6.10	1.5	1.65	23.10	−7.39	−7.18	22.96	66.67	60.98
Forged	6.06	2.5	2.25	6.14	1.58	1.78	21.28	−6.81	−5.30	20.17	63.29	56.18
2024	11.4	5.88	6.12	11.4	2.74	2.75	13.73	−4.39	−5.01	14.15	36.5	36.36

**Table 5:** The material parameters of PZT-5H, epoxy resin and the polymer matching layer

Material: PZT5H Ceramics					
Density (kg/m^3^)	Dielectric constants $\varepsilon_{ij}\;$($\varepsilon_{0}$)		Piezoelectric constants: $e_{ij}$ (C/m^2^)		
ρ	$\varepsilon_{11}^{S}$	$\varepsilon_{33}^{S}$	$e_{15}$	$e_{31}$	$e_{33}$
7500	1704.4	1433.6	17.0345	−6.6228	23.2403
Elastic stiffness constant: $c_{ij}^{E}$ (10^10^ N/m^2^)					
$c_{11}^{E}$	$c_{12}^{E}$	$c_{13}^{E}$	$c_{33}^{E}$	$c_{44}^{E}$	$c_{66}^{E}$
12.7205	8.02122	8.46702	11.7436	2.29885	2.34742
Material: Epoxy resin			Material: Polymer matching layer		
Density (kg/m^3^)	Young’s modulus (GPa)	Poisson’s ratio	Density (kg/m^3^)	Longitudinal wave velocity (m/s)	Shear wave velocity (m/s)
ρ	*E*	σ	ρ	$v_{l}$	$v_{s}$
1150	3	0.35	1150	2650	1100

**Table 6:** Optimized axial dimensions (thicknesses) of the four transducer structures simulated using the finite element method (unit: mm).

Components	Transducer 1	Transducer 2	Transducer 3	Transducer 4
1-3 Composite	9.50	9.50	9.50	9.50
Al Alloy	-	11.40	-	9.80
Mg Alloy	9.50	-	7.24	-
Polymer	-	-	3.20	3.60

## Data Availability

Dataset available on request from the authors.

## References

[1] Shamsa L., Xu T.B. (2023). Underwater communication acoustic transducers: A technology review. Proc. SPIE.

[2] Yang Y., Zhou J., Li M., Hou X., Yang L., Xie L., Wang L., Shen H., Liu X., Lei B. (2025). Underwater acoustics: Current status and future trends. Tech. Acoust..

[3] Tian F.H., Liu Y.M., Ma R.L., Li F., Xu Z., Yang Y.C. (2021). Properties of PMN-PT single crystal piezoelectric material and its application in underwater acoustic transducer. Appl. Acoust..

[4] Minto C. (1999). Low frequency flexural disc sources for high power, compact arrays. Proc. Inst. Acoust..

[5] Yue Y.M., Wu W.H., Hu Z.X., Zhang B.X., Shi T.Y. (2025). Multiobjective optimization design of class VII flextensional transducer with giant magnetostrictive material. IEEE Sens. J..

[6] Dong H.X., Li Y.H., Li X.H., Sang Y.J., Chen Y.Y. (2026). A lightweight and small moving coil underwater acoustic transducer based on the optimal design of magnetic circuit. Sens. Actuators A Phys..

[7] Wang H.W., Wang L.K., Qin L., Li C., Zhang H. (2017). Research on the stacked piezoelectric composites ring transducer. J. Harbin Eng. Univ..

[8] Bian J.C., Wang Y., Liu Z.J., Shen M.J., Zhao H., Sun Y.L., Zhu J.J. (2021). Ultra-wideband underwater acoustic transducer with a gradient impedance matching layer. Appl. Acoust..

[9] Røed E.S., Bring M., Frijlink M., Henriksen A., Tichy F., Åsjord E.M., Hoff L. (2022). Underwater single crystal piezocomposite transducer with extended usable frequency band. Ultrasonics.

[10] Røed E.S., Bring M., Tichy F., Henriksen A., Åsjord E.M., Hoff L. (2022). Optimization of matching layers to extend the usable frequency band for underwater single-crystal piezocomposite transducers. IEEE Trans. Ultrason. Ferroelectr. Freq. Control.

[11] Pyun J.Y., Kim Y.H., Park K.K. (2023). Design of piezoelectric acoustic transducers for underwater applications. Sensors.

[12] Wu S.R., Xie Y.H., Bai F.S., Teng D. (2025). Optimal design and experimental study on a novel relaxor ferroelectric single crystal transducer based on face shear vibration mode. Ultrasonics.

[13] Li H., Deng Z.D., Carlson T.J. (2012). Piezoelectric materials used in underwater acoustic transducers. Sens. Lett..

[14] Aronov B., Brown D.A. (2014). Historical review of underwater acoustic cylindrical transducer development in Russia for sonar arrays. J. Acoust. Soc. Am..

[15] Wang Y.Y., Wang C.Y., Ye R.D., Yin X., Lan Y. (2026). Bandwidth enhancement in relaxor ferroelectric single crystal longitudinal transducers. Acta Acust..

[16] Ji B.C., Hong L.J., Lan Y. (2021). Ultra-wide operation band of the high-frequency underwater acoustic transducer realized by two-layer 1-3 piezoelectric composite. J. Acoust. Soc. Am..

[17] Qin H., Lu H.H., Zhou J.J., Zhang Y. (2023). Effect of thickness on the performance parameters of modified 1–3 piezoelectric composites. Ceram. Int..

[18] Bian J.C., Hu W.X., Zhou B.M. (2018). Multi-matching layer air-coupled piezocomposite ultrasonic transducer. J. Appl. Acoust..

[19] Cannata J.M., Ritter T.A., Chen W.H., Silverman R.H., Shung K.K. (2003). Design of efficient, broadband single-element (20–80 MHz) ultrasonic transducers for medical imaging applications. IEEE Trans. Ultrason. Ferroelectr. Freq. Control.

[20] Fang H.J., Chen Y., Wong C.M., Qiu W.B., Chan H.L.W., Dai J.Y., Li Q., Yan Q.F. (2016). Anodic aluminum oxide–epoxy composite acoustic matching layers for ultrasonic transducer application. Ultrasonics.

[21] Wang C.Y., Lan Y., Cao W.W. (2021). Tonpilz transducer head mass selection based on excitation signal type. Appl. Acoust..

[22] Zhang S.X., Zhang X.N., Zhao C.L., Li J.N., Song Y., Xie C.Y., Tao H.R., Zhang Y., He Y.H., Jiang Y. (2010). Research on an Mg-Zn alloy as a degradable biomaterial. Acta Biomater..

[23] Guo F.F., Wang Y.L., Huang Z.Y., Qiu W.B., Zhang Z.Q., Wang Z., Dong J., Yang B., Cao W.W. (2018). Magnesium alloy matching layer for PMN-PT single crystal transducer applications. IEEE Trans. Ultrason. Ferroelectr. Freq. Control.

[24] Sun Y.H., Tao J.Y., Guo F.F., Wang F.L., Dong J., Jin L., Li S., Huang X. (2023). AZ31B magnesium alloy matching layer for lens-focused piezoelectric transducer application. Ultrasonics.

[25] Sun Y.H., Wang F.L., Dong J., Jin L., Wang F.H., Dong S. (2022). Finite element simulation of array transducer with different matching layer materials. Piezoelectrics Acoustooptics.

[26] Hou J.B., Li D., Liu Z.J., Ji Z.K., Guan S.F., Li C.C., Qiao X.G., Golovin I.S., Zheng M.Y. (2023). Structure-function integrated magnesium alloys and their composites. J. Magnes. Alloys.

[27] Chen X.H., Yuan Y.X., Zhou X., Chen T., Tang A.T., Chen X., Tan J., Moelans N., Eckert J., Pan F.S. (2025). Harnessing the power of α-Mg: Unveiling the matrix phase’s role in Mg alloys from structure to function. J. Magnes. Alloys.

[28] Geshani M.S., Kalayeh P.M., Asadi A.H., Mirzadeh H., Malekan M., Emamy M. (2023). A review of Mg alloys containing long-period stacking ordered (LPSO) structures with insight into the application of friction stir processing. J. Mater. Res. Technol..

[29] Meier J.M. (2022). Towards high strength cast Mg-RE based alloys: Phase diagrams and strengthening mechanisms. J. Magnes. Alloys.

[30] Xu W.L., Yu J.M., Jia L.C., Gao C., Miao Z., Wu G.Q., Li G.J., Zhang Z.M. (2022). Grain refinement impact on the mechanical properties and wear behavior of Mg-9Gd-3Y-2Zn-0.5Zr alloy after decreasing temperature reciprocating upsetting-extrusion. J. Magnes. Alloys.

[31] Barrett C.D., Imandoust A., Oppedal A.L., Inal K., Tschopp M.A., Kadiri E.H. (2017). Effect of grain boundaries on texture formation during dynamic recrystallization of magnesium alloys. Acta Mater..

[32] Ito T., Saito G., Uehigashi N., Mori H. (2016). Mechanical properties of a flame-resistant Mg alloy as a next-generation transportation structural material. Energy Procedia.

[33] Dong S., Yu Q., Jiang Y.Y., Dong J., Wang F.H., Jin L., Ding W.J. (2017). Characteristic cyclic plastic deformation in ZK60 magnesium alloy. Int. J. Plast..

[34] Papadakis E.P. (1968). Ultrasonic attenuation caused by scattering in polycrystalline media. Phys. Acoust..

